# Digital Inequalities of Family Life Information Seeking and Family Well-Being Among Chinese Adults in Hong Kong: A Population Survey

**DOI:** 10.2196/jmir.3386

**Published:** 2014-10-03

**Authors:** Man Ping Wang, Xin Wang, Kasisomayajula Viswanath, Alice Wan, Tai Hing Lam, Sophia S Chan

**Affiliations:** ^1^School of NursingThe University of Hong KongHong KongChina (Hong Kong); ^2^School of Public HealthThe University of Hong KongHong KongChina (Hong Kong); ^3^Department of Social and Behavioral SciencesHarvard School of Public HealthBoston, MAUnited States; ^4^Center for Community-Based ResearchDana-Farber Cancer InstituteBoston, MAUnited States

**Keywords:** socioeconomic factors, information seeking behavior, family health

## Abstract

**Background:**

Inequalities in Internet use and health information seeking are well documented, but less is known about information for family life activities.

**Objective:**

We investigated the social determinants of online family life information seeking behaviors and its associations with family well-being among Chinese adults in Hong Kong.

**Methods:**

A probability-based telephone survey was conducted in 2012 to record family life information seeking behaviors, including frequency of seeking and paying attention to family life information, levels of trust, and perceived usefulness of family life information. Family well-being was assessed using 3 single items on perceived family harmony, happiness, and health, with higher scores indicating greater well-being. Adjusted odds ratios for family life information seeking behaviors by socioeconomic characteristics and lifestyle behaviors, and adjusted beta coefficients for family well-being by family life information seeking behaviors were calculated.

**Results:**

Of 1537 respondents, 57.57% (855/1537) had ever and 26.45% (407/1537) sought monthly family life information through the Internet. Lower educational attainment and household income, smoking, and physical inactivity were associated with less frequent seeking and paying attention (all *P*<.05). Greater perceived family health was associated with more frequent attention (adjusted β=.32, 95% CI.11-.52), greater levels of trust (adjusted β=.28, 95% CI .07-.48), and perceived usefulness (adjusted β=.23, 95% CI .01-.45) of family life information. Frequent attention and higher level of trust were also associated with greater family harmony (adjusted β=.22, 95% CI .002-.41) and happiness (adjusted β=.23, 95% CI .003-.42), respectively.

**Conclusions:**

This is the first study investigating family life information seeking behaviors and suggested inequalities of online family life information seeking behaviors. The association between family life information seeking behavior and family well-being needs to be confirmed in prospective studies.

## Introduction

Communication inequalities, defined as differences in accessing, processing, and acting on information, may be the link between social determinants and health [1]. Diffusion of advanced information and communication technologies (ICTs) in Hong Kong has led to a high rate of Internet penetration (77.9%), but a digital divide (defined as differential access to Internet among different groups of people) [2] between socioeconomic classes [3]. Prevalence of Internet connection among people living in public housing estates (69.3%) and having low monthly household income (<HK$10,000, 38.9%) was much lower than private housing (82.1%) and income >HK$50,000 (98.2%) [3]. A similar phenomenon was observed for computer use and educational gradients (primary or less: 28.4%; secondary: 82.3%; and postsecondary or greater: 96.8%) [3].

Such inequalities on Internet use suggest a widening gap between information haves and have nots as the Internet is increasingly used as a source for information seeking, although offline searching (eg, television and newspaper) remains an important channel for obtaining information. Bridging communication inequalities is pivotal to reduce social inequality [4]. Most studies have focused on digital health communication inequalities and adverse effects across the continuum of health [5-8]. Inequalities in health information communication only represent a part of the digital divide because the Internet is also used for everyday life information seeking (ELIS), which includes information for solving problems encountered in everyday situations [9,10]. ELIS is studied mostly in the field of information science and rarely in behavioral or social science. Therefore, we aimed to extend current research on digital divide to include information on family life activities.

Family life information is considered an important part of family life, and refers to information to strengthen family functioning through improving communication skills, knowledge about developmental tasks, decision-making skills, self-esteem, and interpersonal relationships [11]. Only a few studies have investigated the family information needs for specific topics, such as parenting, childcare, and information needs for sick children [12-14]. Recognizing the importance of providing family life information, particularly for parents with young children, the UK government has set up family information services in all parts of the country to provide comprehensive family-related information particularly for lower socioeconomic groups [15].

The concept of family life information has not been clearly defined. We adopted a broad, simple, and practical definition of family life information seeking behaviors as information related to family communication; relationships with children, partners, and other family members; work-life balance; and emotion and stress management. These components are commonly reported in many Western studies as main factors affecting family well-being [16,17]. Similarly, our qualitative studies exploring the concepts of family well-being in the general public and community leaders in Hong Kong found that health, happiness, and harmony (3Hs) are the 3 major themes of family well-being [18,19]. The concept of the family 3Hs are coherent with the traditional Chinese value on collectivism, but are different from Western individualist culture, which puts more emphasis on personal happiness and independence than family 3Hs [20].

Specifically, this study aimed to investigate socioeconomic and sociodemographic correlates of frequency of seeking, frequency of attention, levels of trust, and perceived usefulness toward online family life information focusing on socioeconomic inequalities. In addition, the associations between family life information seeking behaviors and the family 3Hs were also investigated.

## Methods

### Sampling

Details of research design have been reported elsewhere [6,21]. In brief, as a part of the FAMILY project, the Hong Kong Family and Health Information Trends Survey (FHInTS) was conducted in 2012 using a random telephone-based survey of the general public to monitor opinions and behaviors related to family health and communication [6]. All interviews were conducted by trained interviewers of the Public Opinion Programme from the University of Hong Kong. A 2-stage random sampling method was used. Telephone numbers were retrieved from residential telephone directories that cover approximately 76% of Hong Kong residents [22]. A computer program was used to generate a list of the telephone numbers in random order for interviews. Invalid household numbers, nonresponses, and ineligible households (people aged <18 years or not able to speak Cantonese) were excluded (n=8748). In the second stage, after interviewers introduced the study purpose, adult respondents were asked how many eligible persons were living in the household. All eligible persons were listed and the person with the next birthday closest to the interview day was selected for interview. Each interview took approximately 25 minutes to complete. Among 2080 people with confirmed eligibility, 1537 adults were successfully interviewed yielding a response rate of 73.9% [23]. Ethical approval was granted by the Institutional Review Board (IRB) of the University of Hong Kong/Hospital Authority Hong Kong West Cluster. Verbal informed consent was obtained and recoded verbatim, and the procedure was approved by the IRB.

### Measurement

Definitions of families (family members are those who have relationships through biological, marital, cohabital, or emotional bonding) and family life information seeking (definition as mentioned previously) were presented to the respondents prior to the questions about family life information seeking. Frequency of family life information seeking was assessed by the question: “In the past 12 months, how often have you searched for family life information on the Internet?” with responses of ≥1 time/week, 1-3 times/month, 1 time in several months, rarely, and never/do have not Internet access. Attention to family information was assessed by the question “How often did you pay attention to family life information” with responses of frequently, sometimes, rarely, and never. Trust in family life information was assessed by the question: “What do you think about the online family information sought last time?” with responses of very trustful, partly trustful, neutral, not trustful, and completely not trustful. Perceived usefulness of online family life information was assessed by the question: “Do you think online family life information is useful?” with responses of very useful, partly useful, little useful, and not useful. Perceived family harmony, happiness, and health was assessed using 3 separate questions asking respondents to give a score from 0-10 with a higher score indicating better family well-being. Internal consistency of these 3 items was supported by a satisfactory Cronbach alpha (.84).

As in other similar studies on information seeking [5,24], socioeconomic status (SES) was measured using educational attainment, household monthly income, and employment. Several studies have documented the influence of these SES variables on a variety of health outcomes [25-27]. The responses for SES were based on our previous studies and the Hong Kong census with slight modifications. Education attainment was categorized as primary or less (combining no formal education and primary education), secondary, and tertiary or greater. Monthly household income (in Hong Kong dollars; US $1 = HK$7.8) was categorized as <HK$10,000 (combining <HK$4000 and HK$4000-HK$9999), HK$10,000-HK$19,999, HK$20,000-HK$29,999, HK$30,000-HK$39,999, and ≥HK$40,000. Employment status was categorized as full-time, part-time, self-employed, and unemployed. Health behaviors were correlated with information seeking as found in our previous studies [6]. We assessed the associations of smoking (never, ex-, and current), alcohol drinking (never, ex-, occasional, and monthly), and physical activity (none, 1-3 days/week, ≥4 days/week) with family life information seeking.

### Statistical Analysis

All data were weighted by sex and age from the census data. Inequalities in family life information seeking by sex, age, marital status, and SES indicators were assessed by logistic regression, which yielded adjusted odds ratios (aOR) for family life information seeking. The association between family life information seeking and family well-being was estimated using linear regression (beta coefficients) adjusted for sociodemographic characteristics and health status. All analyses were performed using STATA 10 (StataCorp LP, College Station, TX, USA). A *P* value of <.05 was considered statistically significant.

## Results

Of all respondents (N=1537), 45.71% (703/1537) were male, 73.78% (1123/1522) aged 25-64 years, 61.13% (937/1533) were married, 86.59% (1325/1530) had secondary school educational attainment, and 61.81% (801/1296) had monthly household income ≥HK$ 20,000. Details of socioeconomic status are reported elsewhere [28]. Sample representativeness was supported by small difference in distribution of sex, age, educational attainment, and household income between our sample and the general population (Cohen’s effect size <0.3) [28].

Table 1 shows that one-quarter of respondents (25.8%, 396/1537) sought family life information for recreational purposes followed by information for improving family relationships (17.7%, 272/1537) and communication (15.3%, 235/1537), managing emotional problems and stress (14.0%, 215/1537), and improving work and ability (10.8%, 165/1537). More than half of the respondents (57.57%, 885/1537) had ever sought family life information with 26.45% (407/1537) on a monthly basis. In addition to active seeking, more than two-thirds (69.12%, 1059/1532) of the respondents had ever paid attention to family life information. Only 3.2% (39/1198) reported online family life information as trustful and 10.5% (137/1309) of respondents reported it as very useful.

**Table 1:** Online family life information seeking content, frequency, attention, and trust (N=1537).

Content		Unweighted, n (%)	Weighted, n (%)
Entertainment information		334 (21.73)	396 (25.75)
**Family relationship**		223 (14.51)	272 (17.68)
	Children	177 (11.52)	208 (13.53)
	Couples	104 (6.77)	135 (8.77)
	Relatives	28 (1.82)	37 (2.42)
**Family communication**		199 (12.95)	235 (15.27)
	Emotion and stress management	178 (11.58)	215 (14.02)
	Improvement of self/work-ability	134 (8.72)	165 (10.72)
	Work-life balance	66 (4.29)	87 (5.67)
	Ability of self-independent	59 (3.84)	77 (5.02)
	Others	42 (2.73)	45 (2.93)
**Frequency of seeking**			
	≥1 time(s)/week	98 (6.38)	120 (7.78)
	1-3 times/month	242 (15.76)	287 (18.67)
	<1 time/month	423 (27.54)	478 (31.12)
	Never	773 (50.33)	652 (42.43)
**Attention**			
	Always	189 (12.37)	228 (14.87)
	Sometimes	438 (28.66)	491 (32.06)
	Seldom	316 (20.68)	340 (22.19)
	Never	585 (38.29)	473 (30.88)
**Trust**			
	Trust	38 (3.50)	39 (3.24)
	Some trust	587 (54.00)	657 (54.88)
	Neutral	291 (26.77)	328 (27.38)
	Not trust	150 (13.80)	152 (12.66)
	Absolutely not trust	21 (1.93)	22 (1.83)
**Perceived information usefulness**			
	Very useful	122 (9.78)	137 (10.48)
	Partly useful	625 (50.08)	714 (54.56)
	Little useful	182 (14.58)	186 (14.20)
	Not useful	319 (25.56)	272 (20.77)

Table 2 shows that age was inversely associated with monthly seeking, frequent attention, and perceived usefulness toward family life information (*P* for trend <.001) although no association was observed for trust. Educational attainment was strongly associated with seeking, attention, trust, and perceived usefulness of family life information (all *P* for trend <.008-<.001). Similarly, compared with family income <HK$10,000, higher household income was generally associated with greater odds of seeking, attention, and perceived usefulness toward family life information although nonsignificant associations for the highest income category of ≥HK$30,000 with attention and perceived usefulness were found. Marital and employment statuses were not associated with family life information seeking behaviors. In addition, family income was significantly associated with perceived levels of family health (β=.37, *P*=.04) and harmony (β=.90, *P*=.005).

**Table 2:** Socioeconomic status and online family life information seeking behaviors (N=1537).

Factors		Monthly seeking			Frequent attention		Trust			Perceived usefulness		
		n (%)		aOR (95% CI)^a^	n (%)	aOR (95% CI)^a^	n (%)	aOR (95% CI)^a^		n (%)	aOR (95% CI)^a^
**Sex**											
	Male	198 (28.2)		1	342 (48.9)	1	319 (56.0)	1		407 (66.9)	1
	Female	208 (25.0)		0.90 (0.69-1.19)	376 (45.3)	0.92 (0.71-1.20)	376 (60.1)	1.12 (0.85-1.47)		444 (63.4)	0.76 (0.56-1.02)
**Age (years)**											
	18-24	68 (43.8)		1	102 (65.4)	1	96 (61.8)	1		115 (74.5)	1
	25-44	212 (37.8)		0.69 (0.43-1.12)	361 (64.4)	0.77 (0.46-1.27)	323 (59.4)	1.02 (0.63-1.64)		411 (76.3)	1.40 (0.80-2.46)
	45-64	113 (20.2)		0.38 (0.22-0.65)^d^	225 (40.4)	0.42 (0.24-0.72)^c^	229 (55.3)	0.92 (0.54-1.56)		273 (59.1)	0.85 (0.46-1.57)
	≥65	10 (4.3)		0.09 (0.04-0.25)^d^	26 (10.9)	0.12 (0.06-0.25)^d^	39 (54.3)	1.47 (0.65-3.31)		45 (31.3)	0.28 (0.13-0.60)^c^
	*P* for trend			<0.0001		<0.0001		0.89			<0.0001
**Marital status**											
	Single	185 (38.0)		1		1		1			1
	Married/cohabitated	214 (22.7)		1.02 (0.73-1.43)	308 (63.2)	1.03 (0.74-1.45)	277 (59.6)	1.03 (0.73-1.44)		354 (76.2)	0.77 (0.51-1.14)
	Other	7 (7.4)		0.75 (0.28-1.99)	394 (41.9)	0.67 (0.31-1.46)	399 (57.8)	0.81 (0.36-1.84)		471 (60.6)	0.96 (0.44-2.08)
**Employment status**					16 (15.7)		17 (44.3)			26 (40.4)	
	Full-time	252 (35.2)		1		1		1			1
	Part-time	32 (27.7)		1.18 (0.71-1.97)	425 (59.4)	0.73 (0.45-1.19)	395 (60.5)	0.87 (0.52-1.44)		492 (73.8)	0.95 (0.56-1.61)
	Self-employed	16 (22.1)		0.70 (0.38-1.29)	50 (43.6)	0.79 (0.46-1.37)	51 (54.3)	0.65 (0.38-1.13)		65 (64.3)	0.62 (0.35-1.11)
	Unemployed	107 (16.8)		0.93 (0.65-1.33)	34 (46.3)	0.88 (0.63-1.23)	31 (50.0)	1.07 (0.75-1.52)		39 (59.3)	1.16 (0.80-1.68)
**Education**					210 (33.4)		218 (56.4)			254 (53.7)	
	≤Primary	3 (1.6)		1		1		1			1
	Secondary	170 (23.7)		7.42 (2.38-23.12)^d^	12 (6.0)	5.44 (2.81-10.52)^d^	25 (39.0)	2.23 (1.21-4.12)^b^		26 (22.1)	2.98 (1.72-5.16)^d^
	≥Tertiary	232 (38.2)		11.04 (3.47-35.12)^d^	305 (42.8)	10.69 (5.34-21.43)^d^	310 (55.5)	2.75 (1.44-5.25)^c^		368 (60.6)	6.30 (3.44-11.54)^d^
	*P* for trend			<0.0001	397 (65.5)	<0.0001	357 (62.6)	0.008		455 (78.9)	<0.0001
**Household income (HK$)**											
	≤9999	16 (7.5)		1		1		1			1
	10,000-19,999	78 (28.2)		2.02 (1.09-3.76)^b^	39 (17.8)	1.84 (1.13-2.99)^b^	44 (50.0)	1.26 (0.73-2.16)		56 (40.2)	1.40 (0.85-2.32)
	20,000-29,999	74 (28.8)		1.94 (1.03-3.64)^b^	134 (49.3)	1.84 (1.12-3.02)^b^	122 (54.5)	1.48 (0.85-2.57)		152 (63.6)	1.95 (1.15-3.31)^b^
	≥30,000	194 (35.7)		2.10 (1.14-3.88)^b^	134 (52.6)	1.58 (0.98-2.56)	127 (59.3)	1.55 (0.90-2.65)		162 (72.3)	1.46 (0.88-2.42)
	*P* for trend			0.11		0.42		0.10			0.24

^a^ aOR: adjusted odds ratio; mutually adjusted for the variables in the table.

^b^ *P*<.05.

^c^ *P*<.01.

^d^ *P*<.001.

Compared with never smoking, current smoking was associated with lower aORs of 0.35 (95% CI 0.19-0.64) and 0.58 (95% CI 0.35-0.96) for monthly seeking and perceived usefulness of family life information, respectively (Table 3). Greater level of moderate physical activity (≥4 days per week) was associated with family life information seeking behaviors particularly for monthly seeking (aOR 1.66, 95% CI 1.19-2.32), frequent attention (aOR 1.69, 95% CI 1.64-2.31), and trust (aOR 1.43, 95% CI 1.04-1.96) of family life information.

Greater level of perceived family health was significantly associated with frequent attention (adjusted β=.32, 95% CI .11-.52), trust (adjusted β=.28, 95% CI .07-.48), and perceived usefulness (adjusted β=.23, 95% CI .01-.45) of family life information, and marginally associated with monthly seeking of family life information (adjusted β=.15, 95% CI –.06 to .36) (Table 4). In contrast, only frequent attention was associated with family harmony (adjusted β=.22, 95% CI .02-.41) and trust of the information was associated with family happiness (adjusted β=.23, 95% CI .03-.42).

**Table 3:** Behavioral correlates and family life information seeking behaviors (N=1537).

Health behaviors		Monthly seeking		Frequent attention		Trust			Perceived usefulness	
		n (%)	aOR (95% CI)^a^	n (%)	aOR (95% CI)^a^		n (%)	aOR (95% CI)^a^	n (%)	aOR (95% CI)^a^
**Smoking**										
	Never	367 (28.7)	1	612 (48.0)	1		604 (59.6)	1	743 (67.2)	1
	Ex-smoker	20 (17.8)	0.78 (0.43-1.42)	39 (34.7)	0.97 (0.57-1.64)		42 (59.2)	0.95 (0.55-1.66)	42 (51.3)	0.58 (0.33-1.02)
	Current smoker	17 (11.9)	0.35 (0.19-0.64)^d^	66 (47.5)	1.40 (0.87-2.24)		49 (44.7)	0.72 (0.44-1.17)	63 (53.4)	0.58 (0.35-0.96)^b^
**Drinking**										
	Never	175 (23.4)	1	305 (41.0)	1		310 (57.9)	1	379 (61.7)	1
	Ex-drinker	6 (18.4)	2.30 (0.73-7.21)	8 (27.7)	1.17 (0.41-3.37)		10 (57.8)	0.98 (0.33-2.88)	9 (44.8)	1.40 (0.44-4.48)
	Occasional drinker	64 (26.8)	1.04 (0.71-1.54)	112 (46.7)	0.88 (0.61-1.27)		115 (58.0)	0.93 (0.64-1.35)	139 (65.2)	0.84 (0.56-1.26)
	Monthly drinker	162 (31.3)	1.08 (0.79-1.46)	293 (56.7)	1.06 (0.79-1.42)		260 (58.5)	0.98 (0.73-1.31)	324 (70.4)	0.98 (0.70-1.36)
**Moderate physical activity**									
	None	158 (23.5)	1	281 (42.0)	1		272 (53.1)	1	359 (62.5)	1
	1-3 days/week	116 (30.3)	1.30 (0.94-1.80)	217 (56.6)	1.47 (1.08-2.01)^b^		208 (64.6)	1.53 (1.12-2.11)^c^	251 (73.6)	1.47 (1.03-2.11)^b^
	≥4 days/week	130 (27.3)	1.66 (1.19-2.32)^c^	219 (46.1)	1.69 (1.64-2.31)^d^		214 (59.5)	1.43 (1.04-1.96)^b^	239 (61.4)	1.05 (0.75-1.46)

^a^ aOR: adjusted odds ratio; adjusted for sex, age, marital status, employment, education, household income, and diseases.

^b^ *P*<.05.

^c^ *P*<.01.

^d^ *P*<.001.

**Table 4:** Online family life information seeking behaviors and family well-being (N=1537).

Family information seeking		Family harmony			Family happiness		Family health		
		Mean (SD)		β (95% CI)^a^	Mean (SD)	β (95% CI)^a^	Mean (SD)	β (95% CI)^a^
**Monthly seeking**								
	No	7.6 (1.6)		0	7.4 (1.6)	0	7.2 (1.7)	0
	Yes	7.6 (1.6)		.15 (–.06, .36)	7.4 (1.5)	.04 (–.16, .24)	7.3 (1.6)	.14 (–.07, .35)
**Frequent attention**								
	No	7.6 (1.6)		0	7.3 (1.6)	0	7.1 (1.7)	0
	Yes	7.7 (1.7)		.22 (.02, .41)^b^	7.4 (1.6)	.14 (–.06, .33)	7.4 (1.6)	.32 (.11, .52)^c^
**Trust**								
	No	7.4 (1.6)		0	7.2 (1.6)	0	7.0 (1.7)	0
	Yes	7.6 (1.7)		.14 (–.07, .34)	7.5 (1.5)	.23 (.03, .42)^b^	7.3 (1.6)	.28 (.07, .48)^c^
**Perceived usefulness**								
	No	7.6 (1.7)		0	7.4 (1.7)	0	7.1 (1.8)	0
	Yes	7.6 (1.6)		.14 (–.09, .36)	7.4 (1.5)	.02 (–.20, .23)	7.3 (1.6)	.23 (.01, .45)^b^

^a^ Adjusted for sex, age, marital status, employment, education, household income, and diseases.

^b^ *P*<.05.

^c^ *P*<.01.

## Discussion

This is probably the first study investigating online family life information seeking behaviors including frequency of seeking and attention, level of trust, and perceived usefulness. In our study, prevalence of online family life information seeking (57.6%) was higher than online health information seeking (44.0%) in Hong Kong [29], but was comparable to health information seeking (72%) in the United States [30], suggesting studying family life information seeking behavior is important. Our study has extended the findings of previous studies on parenting and childcare information [13,14], and revealed that a wide range of family life topics were sought through the Internet, including family recreation, communication, and work-life balance. As a part of family life education in Hong Kong [31], basic online family life information is provided by the Social Welfare Department, Education Bureau, and some nongovernmental organizations (NGOs). On the other hand, the private sector generated numerous online platforms for sharing comprehensive and vibrant family life information in an interactive way. However, the quality and accuracy of the information from these websites is unknown.

Although many respondents paid attention to online family life information (69.1%), very few (3.2%) trusted the information, and only some (10.5%) perceived the information as very useful. The quality of online information is always a concern and similar to patterns seen with health information [32]. This suggests that online platforms providing evidence-based and comprehensive family life information are needed in Hong Kong and elsewhere. Online family life information can be provided by large government sectors or reputable NGOs. Similar websites have been established by the UK National Association of Family Information Services as a mandatory service for providing family life information [15]. Western studies have shown that websites providing family life education were well received by various groups of people [33]. Incorporating family life information into social media and mobile Internet devices will substantially increase the penetration and adoption of such information. Providing the information will not only benefit Hong Kong Chinese, but also millions of families in mainland China and beyond.

We found that online family life information seeking behavior was socially patterned with lower levels of educational attainment and income associated with less frequent family life information seeking and attention. The results were consistent with the digital divide of health information seeking in local and international studies [5,6,24]. Lack of Internet access, lower social support, and poor information literacy and skills among disadvantaged groups are documented barriers for seeking online information [34]. Compared with household income, educational attainment was an even stronger factor associating with information seeking, level of trust, and perceived usefulness. This suggests that cognitive skills are more influential on family life information seeking behaviors than physical access to the Internet. Unlike health information, the concept of family life information is more vague and less developed; thus, high cognitive functions are needed to refine searching, understand sophisticated content, and translate information into behaviors. Online family life information seeking was also patterned by age and lifestyle behaviors. Increasing age, current smoking status, and physically inactivity were risk factors for infrequent family life information seeking and lower level of perceived usefulness. Our findings can help campaign planners of family life information seeking to focus resources on these groups.

More importantly, we found that family life information seeking was associated with greater family well-being, particularly perceived family health and harmony, which were also patterned by socioeconomic status. Although our results need to be confirmed in prospective studies, the findings were in-line with benefits of family life education in preventing family problems and improving family functions [35]. Maintaining family well-being is a challenge particularly for families with long working hours, which is typical in Hong Kong (average weekly working 45 hours) [36] comparable to South Korea (44.6 hours) having the longest working hours in countries of Organisation for Economic Co-operation and Development (OECD). Seeking online family life information is convenient and would help these busy families to better prepare for family activities, effectively solve family problems, and demonstrate care of other family members. Future qualitative and quantitative studies are warranted to investigate the underlying mechanisms between family life information seeking and family well-being.

This study has several limitations. First, a cross-sectional design was used and temporality of the associations cannot be confirmed. It is unlikely that family life information seeking would lead to higher educational attainment and income. Nevertheless, greater levels of family well-being may facilitate seeking of family life information. Prospective studies are needed to confirm the associations and test the mediation effects of family life information seeking on the association between socioeconomic status and the family 3Hs. Second, to the best of our knowledge this is the first study to describe the family life information seeking behaviors. Although a simple description of family life information seeking has been provided, we are uncertain about the variation of family life information seeking definitions perceived by the respondents and how this would affect the results. Future studies are needed to better define family life information seeking probably using a qualitative research design and purposive sampling surveys among respondents who may take a more active role in certain activities in the family to understand the detail of family life information seeking behaviors. The family 3Hs was used a proxy of family well-being as supported by our previous qualitative studies [18,19]. Data from another survey also in the FAMILY project [37] showed family 3Hs items were moderately correlated (Pearson *r* ranges .34-.47) with family functioning (APGAR) [38], and resilience (Family Resilience Assessment Scale). However, given the distinct difference of perceptions on family functions between cultures, we are not certain about the generalizability of the association between family life information seeking and the family 3Hs to other countries. Third, although our sample was representative of the general population, the effects of nonresponse bias and problems of decreasing landline telephone coverage on the observed associations were uncertain.

This study is the first to investigate family life information seeking behaviors. The results showed that people with lower SES were less likely to seek and pay attention to online family life information or perceive family life information as useful. The associations of family life information seeking with perceived family health and harmony need to be confirmed by prospective studies.

## Abbreviations

ELIS: everyday life information seeking
FHInTS: Family and Health Information Trends Survey
ICT: information and communication technologies
IRB: Institutional Review Board
NGO: nongovernmental organization
OECD: Organisation for Economic Co-operation and Development
SES: socioeconomic status
